# Projection of Premature Cancer Mortality in Hunan, China, Through 2030: Modeling Study

**DOI:** 10.2196/43967

**Published:** 2023-03-06

**Authors:** Wenqiong Wu, Jing Wang, Xian-zhen Liao, Kekui Xu, Yanhua Zou, Zhaohui Shi, Yingyun Hu, Haifan Xiao, Can Li, Shiyu Cao, Shiyu Wang, Jia Guo, Zhicheng Luo, Mengjiao Liu, Mengyao Xu, Donghui Jin, Mengshi Chen, Zhongxi Fu, Shipeng Yan

**Affiliations:** 1 Department of Radiotherapy Hunan Cancer Hospital Changsha China; 2 Chengdu Center for Disease Control and Prevention Chengdu China; 3 Department of Epidemiology and Health Statistics Xiangya School of Public Health Central South University Changsha China; 4 Department of Cancer Prevention and Control Hunan Cancer Hospital Changsha China; 5 Hunan Provincial Center for Disease Control and Prevention Changsha China

**Keywords:** cancer, forecasting, premature mortality, risk factors, Sustainable Development Goals

## Abstract

**Background:**

The United Nations Sustainable Development Goals for 2030 include reducing premature mortality from noncommunicable diseases by one-third. Although previous modeling studies have predicted premature mortality from noncommunicable diseases, the predictions for cancer and its subcategories are less well understood in China.

**Objective:**

The aim of this study was to project premature cancer mortality of 10 leading cancers in Hunan Province, China, based on various scenarios of risk factor control so as to establish the priority for future interventions.

**Methods:**

We used data collected between 2009 and 2017 from the Hunan cancer registry annual report as empirical data for projections. The population-attributable fraction was used to disaggregate cancer deaths into parts attributable and unattributable to 10 risk factors: smoking, alcohol use, high BMI, diabetes, physical inactivity, low vegetable and fruit intake, high red meat intake, high salt intake, and high ambient fine particulate matter (PM2.5) levels. The unattributable deaths and the risk factors in the baseline scenario were projected using the proportional change model, assuming constant annual change rates through 2030. The comparative risk assessment theory was used in simulated scenarios to reflect how premature mortality would be affected if the targets for risk factor control were achieved by 2030.

**Results:**

The cancer burden in Hunan significantly increased during 2009-2017. If current trends for each risk factor continued to 2030, the total premature deaths from cancers in 2030 would increase to 97,787 in Hunan Province, and the premature mortality (9.74%) would be 44.47% higher than that in 2013 (6.74%). In the combined scenario where all risk factor control targets were achieved, 14.41% of premature cancer mortality among those aged 30-70 years would be avoided compared with the business-as-usual scenario in 2030. Reductions in the prevalence of diabetes, high BMI, ambient PM2.5 levels, and insufficient fruit intake played relatively important roles in decreasing cancer premature mortality. However, the one-third reduction goal would not be achieved for most cancers except gastric cancer.

**Conclusions:**

Existing targets on cancer-related risk factors may have important roles in cancer prevention and control. However, they are not sufficient to achieve the one-third reduction goal in premature cancer mortality in Hunan Province. More aggressive risk control targets should be adopted based on local conditions.

## Introduction

Cancer is a leading cause of death worldwide, accounting for nearly 10 million deaths in 2020 or nearly one in six deaths [1]. In China, cancer affected more than 4.56 million people and caused 3 million deaths in 2020, accounting for 30.2% of all deaths in the world [2]. Furthermore, cancer is a leading cause of disability-adjusted life years (DALYs), accounting for 17.64% of all DALYs in China in 2019, which was nearly double the global value of 9.88% [3]. The high mortality and disability related to cancer pose a major disease burden worldwide. Cancer not only directly affects the lives of those diagnosed but also has its sequelae impact the family, society, and economy. The socioeconomic burden of cancer has been reported to be substantial, which includes both the direct health care costs and the lost productivity through premature mortality of the working population [4,5].

Cancer is a disease commonly believed to be preventable. In a nationwide study on the risk factors of cancer across 31 provinces of mainland China, Chen et al [6] found that nearly half (45.2%) of all cancer deaths could be attributed to 23 potentially controllable risk factors, which were classified into five categories: behavior, diet, metabolism, environment, and infection. This suggests that millions of deaths could be prevented or delayed each year if appropriate strategies were developed to control these modifiable risk factors, which would undoubtedly greatly reduce the cancer burden in China.

In 2016, the United Nations (UN) set a target to achieve a relative one-third reduction from the 2015 level in premature mortality from noncommunicable diseases (NCDs), including cancer, by 2030 in its Sustainable Development Goal (SDG) target 3.4 [7]. In response to UN SDG target 3.4, the Chinese government issued the Healthy China 2030 Plan with disease prevention and longevity improvement as two of its primary goals [8,9]. A series of policies targeting cancer prevention and treatment was introduced by the government to reduce the burden of premature cancer death in an effort to accomplish SDG target 3.4 of a one-third reduction in premature mortality of NCDs overall. However, considering the vast territory in China and substantial regional differences in socioeconomic and hygienic conditions, the ability to meet SDG target 3.4 will vary greatly across the provinces. Therefore, there is an urgent need for China to implement tailored, region-specific cancer control measures based on reliable data and valid evidence from studies focusing on the local populations.

Hunan Province, located in the central-south of China, is one of the most populous provinces with over 73 million residents. The gross domestic product of Hunan Province was 4.18 trillion yuan (approximately US $606.03 billion) in 2020, ranking 9th among 32 provincial administrative divisions in mainland China. Cancer incidence and mortality rates in Hunan Province in 2018 were 248.24/100,000 and 154.50/100,000, respectively, representing a medium level across the nation [10]. The burdens of years of life lost and DALYs of cancer in Hunan Province were reported to be significantly higher than the national averages [11]. Of note, due to the special local lifestyle habits, Hunan Province has a much higher burden of certain cancer types than other regions, such as oral cavity cancer and nasopharynx cancer, with obviously higher incidences and mortality than national average levels [12,13].

Since publication of the World Health Organization (WHO) Global Monitoring Framework in 2013 [14], several modeling analyses have been conducted to examine the effects of selected risk factor control on reducing NCD mortality under different scenarios worldwide. Kontis et al [15] set six risk factor scenarios based on the Global Monitoring Framework, and estimated their impact on global and regional NCD mortality between 2010 and 2025 using a time-based, population impact fraction formula. They found that achieving all of these targets could efficiently reduce premature mortality from the four main NCDs, including cancers, by nearly 25% globally and in some specific regions [15]. Su et al [16] used an age-period-cohort model for prediction, which suggested that targets of reducing tobacco use could be more ambitious in Taiwan to meet the goal of a 25% reduction in premature cardiovascular disease mortality. Other studies indicated that the risk factor targets recommended by the WHO would be sufficient to help achieve the goal of a one-third reduction in premature mortality for all NCDs combined, but not for certain major subdivisions such as cancers [17,18].

In general, most previous modeling studies focused on overall NCDs at a national level, and there is limited evidence on cancer and its subcategories at a local provincial level. Moreover, the WHO’s voluntary global targets did not include dietary and environmental factors, which are known to be important risk factors for cancer, and it remains unknown whether and how control of these factors may help with cancer prevention.

In light of such research gaps, we performed this study to project premature mortality from cancer in Hunan Province through 2030 under different risk factor control scenarios. Specifically, we projected whether SDG target 3.4 can be met for cancer prevention in Hunan Province and how many deaths from cancers can be prevented if all selected risk factors were controlled. The risk factors were selected based on the Global Monitoring Framework [14] while adding dietary and environmental factors. Projecting future premature mortality under various risk factor control scenarios is crucial for informing decision-making on cancer control and allocating the limited clinical and public health resources to curb the increasing deaths caused by cancer. Our results will help policy makers better formulate priorities for interventions that focus on these risk factors for cancer prevention and control.

## Methods

### Ethics Approval

This analysis centered on publicly available data with no identifiable information on the subjects studied. Therefore, research ethics board approval was not required for this study.

### Selection of Cancer Sites and Related Risk Factors

Cancer sites were selected based on the rank of cancer deaths in the Hunan cancer registry annual report series over the past 10 years, while also taking into account endemic cancer types associated with special local lifestyle habits in Hunan, such as oral cavity cancer and nasopharynx cancer. Finally, 10 leading subcategories were selected, including cancers of the lung, esophagus, liver, stomach, pancreas, prostate, breast, colorectum, oral cavity, and nasopharynx.

Correspondingly, risk factors were selected based on the following criteria: (1) causally associated with cancers as evidenced by the latest Global Burden of Disease (GBD) study [3] or the Continuous Update Project (CUP) Expert Report (low vegetable and fruit intake were included due to consistent evidence showing their causal associations with cancers [19]); (2) available data of exposure levels from officially representative surveys or epidemiological studies; (3) potentially modifiable by available interventions; and (4) recognized as one of the leading global or national causes of disease burden. Ultimately, 10 risk factors were selected, including smoking, alcohol use, physical inactivity, high BMI, low vegetable intake, low fruit intake, high red meat intake, high salt intake, diabetes, and fine particulate matter (PM2.5). Details of cancer sites and related risk factors are shown in Multimedia Appendix 1.

### Data Sources

The National Program of Cancer Registries (NPCR), launched in 2008, is responsible for the collection, evaluation, and publication of cancer data in China. In Hunan Province, 70.4% of cancer patients have registered in the NPCR to date [20], and all cancer cases are coded according to the International Classification of Diseases, 10th Revision. The details of data collection, management, and analysis for cancer registration in China have been described elsewhere [21]. In this study, we extracted the cause-specific cancer death rates, along with annual population data, between 2009 and 2017 from Hunan Statistical Yearbook 2020 to estimate the number of cancer deaths throughout the province. Information on risk factors was mainly obtained from the Chinese Chronic Disease and Risk Factor Surveillance (CCDRFS) survey, which is an ongoing, nationally representative survey involving a set of standard questionnaire interviews, physical examinations, and biological sampling [22]. In this analysis, we used exposure data in the years 2010 and 2018 to represent 2009 and 2017, respectively, due to a lack of investigations. Regarding PM2.5, since systematic monitoring in China did not start until 2013, we extracted data for Hunan from China Regional Estimates (V4.CH.03) of the Saint Louis University Atmospheric Composition Analysis Group, which applied a geographically weighted regression to calibrate regional PM2.5 concentrations through ground-based observations. Overall, population exposures to these risk factors were measured using metrics related to their variable types. For instance, smoking status and alcohol use were measured as the prevalence of people exposed, while dietary factors were measured as continuous variables. The specific details of risk factor measurements are shown in Multimedia Appendix 1.

To ensure data quality, the relative risk (RR) estimates for risk-cancer pairs were preferentially derived from summary results published by the GBD series and the CUP Expert Report. If these were not available, priority was given to meta-analyses or systematic reviews conducted in China or Asia. Studies that provided RRs on our predefined metrics were preferred and estimates for both genders were assumed to be equal if no separate values were available.

### Constructing Risk Factor Scenarios

Based on the Global Monitoring Framework and Healthy China 2030, we constructed 12 separate scenarios of risk factor exposure for the year 2030. Among them, the baseline scenario projected cancer mortality to 2030 assuming that all risk factors continue to follow current trends (see Multimedia Appendix 2 for details on the risk factor exposure estimation). The baseline scenario was simulated using a proportional change model based on the historical trends of risk factors. The other 11 scenarios projected cancer mortality assuming that each of the 10 risk factors achieved the target of domestic or foreign control standards, both separately and in combination. Specifically, the first 10 scenarios were modeled when each risk factor achieved its target, respectively, and the last scenario was modeled when all 10 risk factors achieved their targets. Targets for red meat, vegetable, and fruit intake were set according to the currently available literature. Given that the current annual average PM2.5 concentration in Hunan Province has reached grade II of China’s air quality standards, we used grade I of 15 μg/m^3^ as the target for PM2.5, which was also consistent with phase III of the WHO’s air quality guidelines [23]. Targets for other risk factors were set according to the WHO’s voluntary global targets [14]. Among the 10 risk factors, categorical exposures were lowered directly to the target levels in 2030, whereas targets for continuous exposures were established by shifting the population distributions left or right, assuming a constant distribution for each age-sex stratum. Exceptionally, metrics for exposures such as BMI and diabetes were held constant. Table 1 provides the details of the risk factors for each of the 12 scenarios.

**Table 1:** Scenario specifications in risk factor exposure projection according to the World Health Organization Global Monitoring Framework.

Scenario	Scenario specification
Natural trend	Age- and sex-specific risk factor exposures were projected assuming the annual change rate remained similar to that between 2009 and 2017.
Harmful alcohol use	Age- and sex-specific prevalence of harmful alcohol use is reduced relatively by 10% from the 2013 level. All other risk factors follow the natural trends.
Smoking	Age- and sex-specific prevalence of smoking in 2030 is reduced relatively by 30% from the 2013 level. All other risk factors follow the natural trends.
Physical inactivity	Age- and sex-specific prevalence of physical inactivity in 2030 is 10% relatively lower than that in 2013. All other risk factors follow the natural trends.
Diabetes	Age- and sex-specific prevalence of diabetes in 2030 is the same as in 2013. All other risk factors follow the natural trends.
High BMI	Age- and sex-specific distributions of BMI in 2030 are the same as in 2013. All other risk factors follow the natural trends.
Low vegetable intake	Age- and sex-specific prevalence of low vegetable intake in 2030 is reduced relatively by 30% from the 2013 level. All other risk factors follow the natural trends.
Low fruit intake	Age- and sex-specific prevalence of low fruit intake in 2030 is reduced relatively by 30% from the 2013 level. All other risk factors follow the natural trends.
High red meat intake	Age- and sex-specific prevalence of high red meat intake in 2030 is reduced relatively by 30% from the 2013 level. All other risk factors follow the natural trends.
High salt intake	Age- and sex-specific mean population salt intake in 2030 is reduced relatively by 30% from the 2013 level. All other risk factors follow the natural trends.
PM2.5^a^	The annually averaged PM2.5 concentration in 2030 is reduced to 15 μg/m^3^, according to grade I of air quality standard GB3095-2012. All other risk factors follow the natural trends.
All targets are achieved in 2030	All targets described above are achieved in 2030.

^a^PM2.5: fine particulate matter.

### Projection of Cancer Mortality

Our analysis was focused on examining premature mortality under 12 different scenarios. Consistent with the global documents, we defined premature cancer mortality as the probability of dying from cancers between the ages of 30 and 70 years [24]. To predict mortality for 2030, we considered the annual total deaths to be a function of two separate projections: a trend of deaths potentially driven by the selected risk factors and a business-as-usual (BAU) trend unattributable to these risk factors. Based on these trends, several steps were involved. First, according to the comparative risk assessment [25], we used the population-attributable fraction (PAF) to divide annual deaths into two parts that were attributable or unattributable to the specified risk factors. The PAF estimates the fraction of health outcomes that would be eliminated if the exposures were altered to ideally counterfactual distributions, and different formulas were applied for discrete and continuous variables in the calculations [25]. Second, we projected the unattributable deaths using the proportional change model, assuming that changes in this part would continue to follow the trends observed between 2009 and 2017. Risk factors in the baseline scenario were projected in the same manner. For the other 11 scenarios, the targeted change of each factor was distributed evenly between 2017 and 2030 to obtain an annual PAF value. Third, the total deaths for different scenarios in 2030 could be estimated using the unattributable deaths and PAFs, and the premature mortality could be calculated using age-specific death rates (in 5-year age groups) with a life table method. The levels in 2013 were considered as the baseline for calculations of relative reductions in this study. Analyses for PAF were performed using MATLAB 7.0, while other data were analyzed in SAS 9.4. Details of the analysis methods are shown in Multimedia Appendix 3.

## Results

### Premature Mortality From Cancers 2009-2017

Table 2 shows the estimated number of cancer deaths and mortality by gender in Hunan Province, China, from 2009 to 2017. The number of cancer deaths increased each year for both genders. The age-standardized mortality rates showed an increasing trend, with an annual percentage change of 1.60% for the total population, 2.19% for men, and 1.65% for women.

The premature mortality rates from selected cancers are shown in Table 3 and the trend analysis results are shown in Table 4. The average annual percentage change (AAPC) showed a significant increasing trend for all cancers combined (2.61%), with the largest AAPC occurring in oral cavity cancer (17.26%), followed by esophageal cancer (7.60%), colorectal cancer (7.13%), pancreatic cancer (7.10%), and lung cancer (4.06%). For the other cancers, the premature mortality remained stable with nonsignificant AAPC values, although a decreasing trend was observed in stomach cancer (–1.16%) and liver cancer (–1.92%), while an increasing trend was observed in nasopharynx cancer (2.00%), prostate cancer (11.91%), and breast cancer (4.82%).

**Table 2:** Estimated cancer deaths and mortality rates in Hunan Province, China, 2009-2017.

Year	Total			Men				Women		
	Deaths, n	Mortality rate (1/100,000)	Standardized mortality rate (1/100,000)	Deaths, n	Mortality rate (1/100,000)	Standardized mortality rate (1/100,000)	Deaths, n		Mortality rate (1/100,000)	Standardized mortality rate (1/100,000)
2009	81,159	117.56	89.53	50,480	140.88	108.62	30,679		92.49	69.51
2010	83,332	127.03	87.13	53,938	159.69	110.53	29,394		92.07	62.85
2011	88,269	123.61	83.53	56,536	152.84	104.19	31,733		92.34	62.21
2012	95,044	132.08	91.14	62,883	168.78	117.78	32,161		93.11	63.74
2013	96,173	133.84	87.14	63,640	171.43	112.76	32,533		94.71	61.18
2014	102,821	142.47	92.58	67,338	180.00	119.36	35,483		102.51	65.26
2015	102,310	140.91	91.50	66,543	176.93	117.34	35,767		102.75	65.11
2016	108,097	147.48	97.57	70,590	185.88	125.61	37,507		106.52	68.81
2017	111,719	152.91	99.38	72,708	192.26	128.18	39,011		111.00	69.91
APC^a^ (95%CI)	N/A^b^	3.13 (2.50-3.80)	1.60 (0.50-2.70)	N/A	3.43 (2.40-4.50)	2.19 (0.60-3.40)	N/A		2.54 (1.70-3.30)	1.65 (0.60-2.70)
*P* value	N/A	<.001	.011	N/A	<.001	.002	N/A		<.001	.007

^a^APC: annual percentage change.

^b^N/A: not applicable.

**Table 3:** Premature mortality (%) from selected cancers in Hunan Province, China, 2009-2017.^a^

Year	Lung cancer	Gastric cancer	Liver cancer	Colorectal cancer	Esophageal cancer	Pancreatic cancer	Nasopharynx cancer	Oral cavity cancer	Prostate cancer	Breast cancer	All cancers
2009	1.95	0.66	1.36	0.44	0.16	0.11	0.18	0.05	0.02	0.65	6.64
2010	2.02	0.60	1.64	0.34	0.16	0.10	0.19	0.04	0.07	0.32	6.53
2011	1.87	0.50	1.63	0.36	0.23	0.08	0.25	0.02	0.03	0.36	6.26
2012	2.25	0.55	1.46	0.54	0.29	0.12	0.28	0.05	0.03	0.49	7.06
2013	2.15	0.53	1.13	0.48	0.24	0.13	0.23	0.08	0.07	0.44	6.74
2014	2.42	0.57	1.27	0.55	0.25	0.13	0.23	0.09	0.06	0.55	7.31
2015	2.41	0.48	1.24	0.56	0.26	0.14	0.22	0.09	0.07	0.57	7.22
2016	2.55	0.56	1.35	0.62	0.29	0.16	0.23	0.11	0.07	0.62	7.83
2017	2.57	0.59	1.39	0.63	0.26	0.17	0.21	0.11	0.07	0.62	7.88

^a^Premature mortality is the probability of dying between the ages of 30 and 70 years from a specific cause that was calculated using the life table method.

**Table 4:** Annual percentage change (APC) in premature mortality from selected cancers in Hunan Province, China, 2009-2017.

Cancer type			APC (95% CI)		*P* value
Lung			4.06 (2.50 to 5.60)		<.001
Gastric			–1.16 (–4.00 to 1.80)		.38
Liver			–1.92 (–5.40 to 1.70)		.24
Colorectal			7.13 (3.00 to 11.50)		.005
**Esophageal**					
	2009-2012	21.59 (2.20 to 44.60)		.04	
	2012-2017	0.03 (–7.50 to 8.00)		.99	
	Average	7.60 (1.60 to 13.90)		<.001	
Pancreatic			7.10 (2.90 to 11.40)		.005
**Nasopharynx**					
	2009-2012	13.91 (3.20 to 25.70)		.02	
	2012-2017	–4.59 (–8.70 to –0.30)		.04	
	Average	2.00 (–1.30 to 5.30)		.20	
Oral cavity			17.26 (3.60 to 32.80)		.02
Prostate			11.91 (–1.30 to 26.80)		.07
Breast			4.82 (–2.40 to 12.60)		.17
All cancers			2.61 (1.40 to 3.80)		.001

### Premature Mortality in the Baseline Scenario by 2030

Table 5 shows a comparison of premature deaths and mortality between 2013 and projected for 2030 in the baseline scenario where all risk factors continue their current trends. In 2030, an estimated 97,787 people would die prematurely from all cancers, with a premature mortality rate of 9.74%, which was 44.47% higher than that in 2013 (Table 5). For the number of premature deaths, lung cancer was expected to account for the largest proportion of total cancer deaths (29,337 deaths), followed by liver cancer (15,545 deaths), colorectal cancer (12,342 deaths), oral cavity cancer (7470 deaths), and esophageal cancer (5977 deaths). All cancers showed increases in the number of premature deaths except for gastric cancer, which was expected to decrease from 4104 in 2013 to 3053 in 2030. The mortality rate of all cancers was estimated to increase by 81.55% from 143.54 to 260.59 per 100,000 people. All cancers exhibited increasing trends in mortality rate except for gastric cancer, which was expected to decrease by 24.62% from 2013 to 2030.

The premature mortality rates for all cancers and each subcategory were consistently higher in men than in women, with differences of more than 5-fold (Table 5). For all cancers, relative increments of 61.47% and 13.46% were observed among men and women, respectively. For men, all cancers showed substantial increases in premature mortality, ranging from 38.25% for lung cancer to 1194.33% for oral cavity cancer, except for gastric cancer, which showed a decreasing trend (–32.51%). For women, most cancers showed increases in premature mortality, ranging from 14.04% for lung cancer to 158.84% for colorectal cancer, except for esophageal cancer (–64.61%), gastric cancer (–52.16%), pancreatic cancer (–45.08%), and nasopharynx cancer (–21.86%). Despite significant gender discrepancies, premature mortality for the whole population generally showed an increasing trend, with the greatest increase occurring in oral cavity cancer (971.53%).

**Table 5:** Deaths and premature mortality of main cancers for people aged 30-70 years in 2013 and projections for 2030 if risk factor trends continue in Hunan Province, China.

Disease		2013			2030				Percent change		
		Premature deaths, n	Mortality rate (1/100,000)	Premature mortality (%)	Premature deaths, n	Mortality rate (1/100,000)	Premature mortality (%)	Premature deaths		Mortality rate	Premature mortality
**Men**											
	Total	36,886	188.18	8.82	71,920	382.42	14.24	94.98		103.22	61.47
	Lung cancer	12,700	64.79	3.28	22,599	120.17	4.54	77.95		85.47	38.35
	Gastric cancer	2785	14.21	0.69	2290	12.17	0.46	–17.78		–14.30	–32.51
	Liver cancer	7373	37.61	1.71	12,360	65.72	2.61	67.64		74.72	52.34
	Colorectal cancer	2405	12.27	0.60	7539	40.09	1.60	213.49		226.74	164.82
	Esophageal cancer	1624	8.29	0.43	5916	31.46	1.35	264.19		279.58	210.91
	Pancreatic cancer	591	3.01	0.15	1414	7.52	0.28	139.34		149.45	82.95
	Nasopharynx cancer	1508	7.70	0.34	2852	15.16	0.63	89.07		97.06	82.95
	Oral cavity cancer	591	3.01	0.13	7255	38.58	1.71	1128.22		1180.13	1194.33
	Prostate cancer	253	1.29	0.07	2504	13.31	0.52	889.01		930.81	592.15
	Other cancers	7046	23.68	1.72	7193	38.25	1.46	2.08		61.53	–14.85
**Women**											
	Total	17,687	97.25	4.51	25,867	138.19	5.11	46.25		42.10	13.46
	Lung cancer	3409	18.75	0.95	6738	36.00	1.38	97.63		92.02	44.93
	Gastric cancer	1319	7.25	0.36	763	4.08	0.17	–42.13		–43.78	–52.16
	Liver cancer	1928	10.60	0.51	3185	17.01	0.59	65.19		60.50	14.04
	Colorectal cancer	1380	7.59	0.36	4803	25.66	0.92	248.04		238.15	158.84
	Esophageal cancer	112	0.61	0.04	61	0.33	0.01	–45.30		–46.86	–64.61
	Pancreatic cancer	365	2.01	0.10	271	1.45	0.06	–25.73		–27.84	–45.08
	Nasopharynx cancer	497	2.73	0.11	400	2.14	0.09	–19.52		–21.81	–21.86
	Oral cavity cancer	101	0.56	0.03	215	1.15	0.04	112.05		106.02	55.81
	Breast cancer	1827	10.04	0.44	3185	17.01	0.69	74.37		69.42	57.56
	Other cancers	6748	29.51	1.69	6245	33.36	1.27	–7.45		13.03	–24.88
**Both genders**											
	Total	54,572	143.54	6.74	97,787	260.59	9.74	79.19		81.55	44.47
	Lung cancer	16,109	42.18	2.15	29,337	78.18	2.96	82.12		85.33	37.57
	Gastric cancer	4104	10.79	0.53	3053	8.14	0.32	–25.61		–24.62	–39.93
	Liver cancer	9301	24.35	1.13	15,545	41.42	1.59	67.13		70.11	40.72
	Colorectal cancer	3785	9.97	0.48	12,342	32.89	1.25	226.09		229.86	158.76
	Esophageal cancer	1736	4.52	0.24	5977	15.93	0.68	244.29		252.39	182.06
	Pancreatic cancer	956	2.52	0.13	1685	4.49	0.17	76.26		78.18	30.58
	Nasopharynx cancer	2006	5.26	0.23	3252	8.67	0.35	62.15		64.77	53.36
	Oral cavity cancer	692	1.81	0.08	7470	19.91	0.87	979.24		1001.08	971.53
	Prostate cancer	253	1.29	0.07	2504	13.31	0.52	889.01		930.81	592.15
	Breast cancer	1827	10.04	0.44	3185	17.01	0.69	74.37		69.42	57.56
	Other cancers	13,794	26.54	1.70	13,438	35.81	1.36	–2.58		34.91	–19.87

### Premature Deaths Avoided and Premature Mortality in Multiple Scenarios

Table 6 shows the projected premature deaths and mortality in 2030 with all risk factors under control, which was further compared with the rates for 2013 and the baseline scenario. Compared with the baseline scenario where all risk factors continue their current trends, 14.90% of premature deaths from cancers would be avoided if all risk factor targets were achieved. Lung cancer had the largest decrease in the number of cancer deaths compared to the baseline scenario (–27.30%), followed by colorectal cancer (–19.88%), esophageal cancer (–17.73%), and breast cancer (–14.08%). A further comparison of avoided cancer deaths by gender showed that men (–15.53%) benefited much more through these combined risk factor control targets than women (–13.16%).

**Figure 1:**
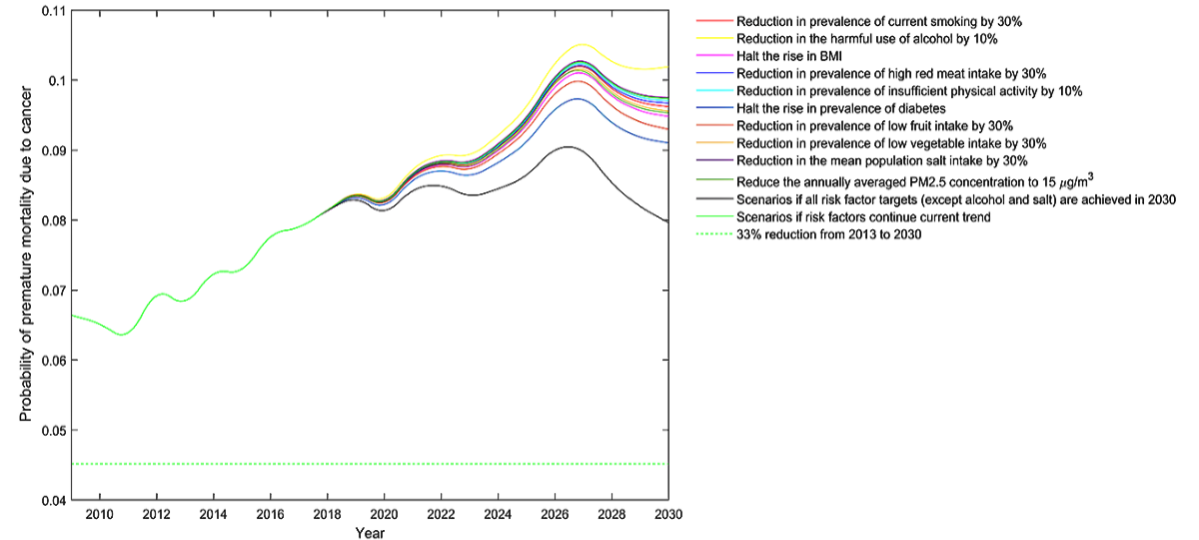
Probability of premature death due to cancers for people between ages 30 and 70 years in Hunan Province, China.

The modeling scenarios seek to avert one-third of premature mortality by 2030. However, this goal is hard to accomplish. For all cancers combined, premature mortality among the total population was expected to increase by 23.65% compared to the baseline year of 2013, even if all risk factor control targets were reached by 2030 (Table 6, Figure 1).

For subcategories, all cancers showed increases in premature mortality compared with that in 2013 in the combined risk factor control target-achieved scenarios, except for gastric cancer with a decrease of 41.63% in the total population (34.45% for men and 53.40% for women). However, it should be noted that the premature deaths and mortality of gastric cancer would still decrease substantially even if all risk factors continue their current trends, as shown in the baseline scenario. A decrease in premature mortality was also found for women in esophageal cancer (72.63%), pancreatic cancer (48.71%), and nasopharynx cancer (29.44%) under combined target–achieved scenarios. Although the combined risk factor control targets failed to achieve the one-third reduction of the cancer mortality rate set by the UN, they could still lead to notable decreases in premature mortality compared with the baseline scenario in 2030.

**Figure 2:**
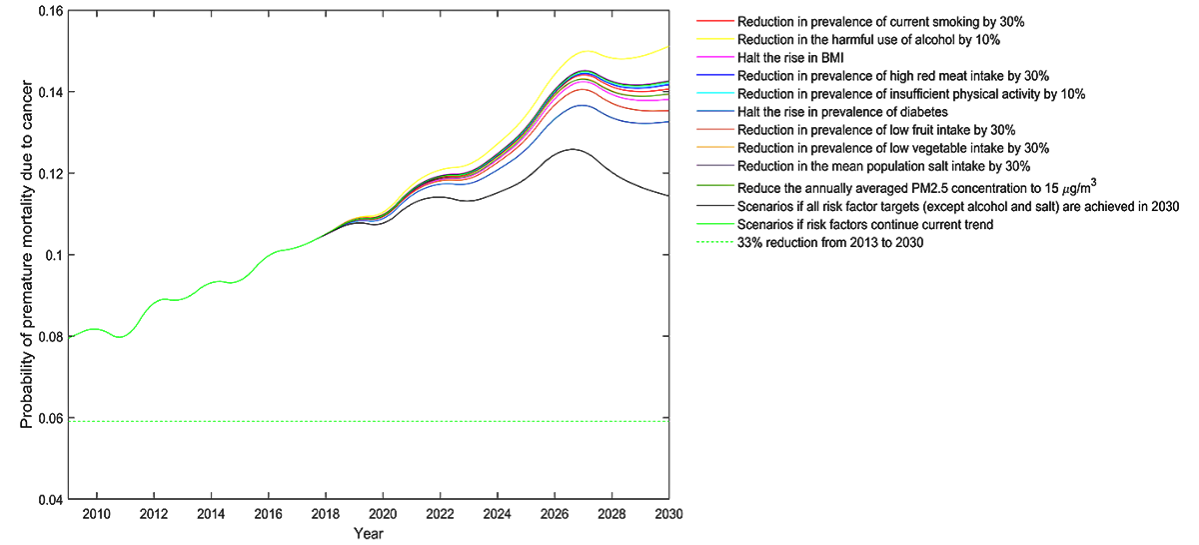
Probability of premature death due to cancers in men aged 30-70 years in Hunan Province, China.

**Figure 3:**
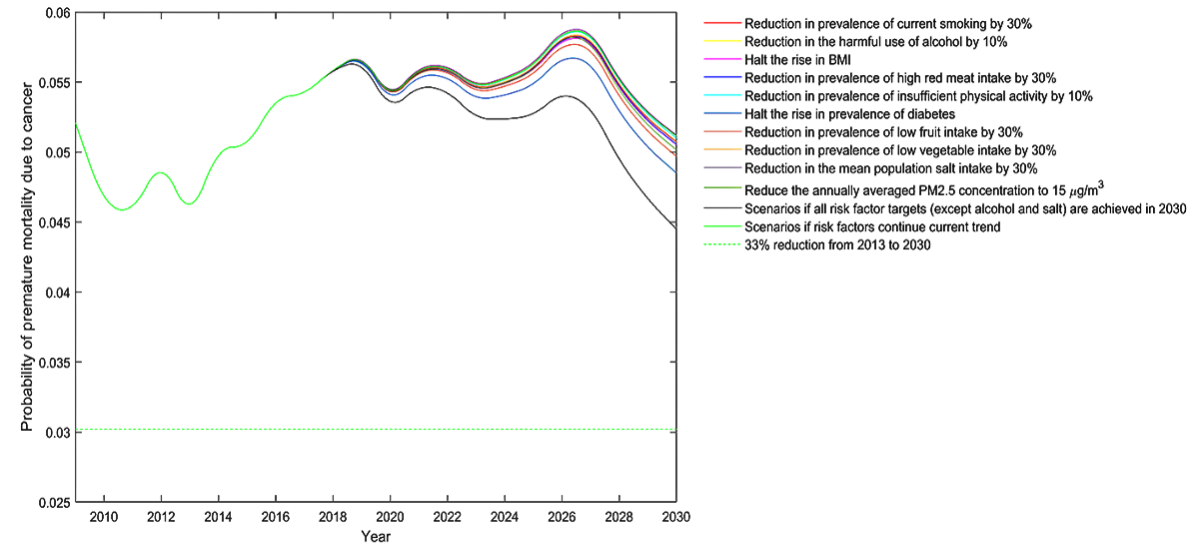
Probability of premature death due to cancers in women aged 30-70 years in Hunan Province, China.

Moreover, the impact on cancer premature mortality varied substantially across different risk factors. For all cancers combined, diabetes and low fruit intake were the top two leading risk factors of cancer premature mortality for both genders. For instance, halting the rise in the prevalence of diabetes may contribute to nearly half of the reductions in cancer premature mortality for both genders (Figure 1). The impact of other risk factors on cancer premature mortality depended on gender. A halt in the rise of BMI had the third-largest impact for men, whereas for women (Figure 2), it was the reduction in ambient PM2.5 levels (Figure 3). For risk factors such as smoking, high red meat intake, and physical inactivity, the predefined risk factor control targets seemed to be insufficient to have pronounced benefits on cancer premature mortality. For risk factors such as harmful alcohol use and high salt intake, the predefined control targets set by the WHO showed much smaller reductions in mortality than the baseline BAU scenario, indicating the need for setting more ambitious targets. In all simulated scenarios, men were projected to have greater gains than women for all cancers after risk factor controls. Scenario projections by gender are shown in Multimedia Appendix 4.

**Table 6:** Premature cancer mortality for people aged 30-70 years in 2030 if all risk factor targets are achieved in Hunan Province, China, and the comparison with baseline values.

Disease		2030 (if all risk factor targets are achieved)			Percent change compared with baseline in 2013, %			Percent change compared with baseline in 2030, %	
		Deaths, n	Premature mortality, %	Deaths		Premature mortality	Deaths		Premature mortality
**Men**									
	Total	60,750	12.15	64.70		37.71	–15.53		–14.72
	Lung cancer	16,207	3.26	27.62		–0.69	–28.28		–28.22
	Gastric cancer	2220	0.45	–20.29		–34.45	–3.05		–2.88
	Liver cancer	10,782	2.25	46.24		31.48	–12.76		–13.69
	Colorectal cancer	5956	1.27	147.66		109.73	–21.00		–20.80
	Esophageal cancer	4870	1.11	199.82		156.42	–17.67		–17.53
	Pancreatic cancer	1267	0.25	114.53		62.42	–10.36		–11.22
	Nasopharynx cancer	2794	0.61	85.23		79.30	–2.03		–2.00
	Oral cavity cancer	6878	1.62	1064.35		1126.49	–5.20		–5.24
	Prostate cancer	2429	0.50	859.36		572.08	–3.00		–2.90
	Other cancers	7193	1.46	2.08		–14.85	0		0
**Women**									
	Total	22,463	4.46	27.00		–1.09	–13.16		–12.83
	Lung cancer	5120	1.05	50.17		10.71	–24.01		–23.61
	Gastric cancer	743	0.17	–43.68		–53.40	–2.68		–2.59
	Liver cancer	2811	0.51	45.80		–0.45	–11.74		–12.71
	Colorectal cancer	3932	0.76	184.92		112.25	–18.14		–18.00
	Esophageal cancer	47	0.01	–57.72		–72.63	–22.70		–22.66
	Pancreatic cancer	253	0.05	–30.66		–48.71	–6.65		–6.60
	Nasopharynx cancer	360	0.08	–27.61		–29.44	–10.05		–9.71
	Oral cavity cancer	186	0.04	83.38		35.34	–13.52		–13.14
	Breast cancer	2737	0.59	49.83		35.58	–14.08		–13.95
	Other cancers	6245	1.27	–7.45		–24.88	0		0
**Both genders**									
	Total	83,213	8.33	52.48		23.65	–14.90		–14.41
	Lung cancer	21,327	2.15	32.39		0.11	–27.30		–27.23
	Gastric cancer	2963	0.31	–27.81		–41.63	–2.96		–2.82
	Liver cancer	13,593	1.37	46.15		21.61	–12.55		–13.58
	Colorectal cancer	9888	1.00	161.25		107.52	–19.88		–19.80
	Esophageal cancer	4917	0.56	183.26		132.30	–17.73		–17.64
	Pancreatic cancer	1520	0.15	59.05		16.93	–9.77		–10.45
	Nasopharynx cancer	3154	0.34	57.26		48.79	–3.01		–2.98
	Oral cavity cancer	7064	0.82	920.53		912.83	–5.44		–5.48
	Prostate cancer	2429	0.50	859.36		572.08	–3.00		–2.90
	Breast cancer	2737	0.59	49.83		35.58	–14.08		–13.95
	Other cancers	13,438	1.36	–2.58		–19.87	0		0

## Discussion

### Principal Findings

In this study, we projected premature mortality from 10 leading cancers in Hunan Province under 12 different risk factor control scenarios in 2030 and evaluated whether SDG target 3.4 can be met for cancer prevention. The results suggest that the one-third reduction goal in premature cancer mortality would not be achieved in Hunan, even if all related risk factor control targets were reached by 2030. This finding is similar to that of a previous study conducted by Li et al [17], showing that achieving all related risk targets would lead to a one-third reduction for all NCDs combined, but not for cancers, in China. These findings suggest that meeting SDG target 3.4 for cancers would require extra efforts from both national and local governments. Notably, Li et al [17] only included six risk factors, in which merely three (ie, smoking, BMI, and physical inactivity) were modeled in the projection of cancer burdens. It seems difficult to achieve dramatic reductions in premature cancer mortality by only controlling for a limited number of risk factors; thus, further inclusion of more risk factors is needed for simulation in the whole country. Although our study contained a wider range of risk factors, the predicted premature cancer mortality in Hunan Province failed to reduce by one-third in 2030, which may be due to the combined effect of several factors such as the conservativeness of some risk factor targets and population aging. According to Chen et al [26], the proportion of people aged over 55 years is expected to increase to more than 30% in Hunan in 2030 [26]. In addition, epidemiological studies have shown that cancer mortality is significantly higher in people over 55 years old than in other age groups, and approximately 27% of cancer deaths were attributed to population aging in China in 2019 [10,21,26], which makes it more difficult to reverse the status quo. Hence, more stringent targets on key risk factors may be required in Hunan.

As for various cancer subcategories, it is interesting that risk factor control targets appeared to generate relatively minor additional benefits for cancers that had already experienced dramatic reductions under the BAU scenario. For other cancers that had not experienced reductions under the BAU scenario, the joint control of all related risk factors may generate larger additional benefits. For instance, the control of all five modifiable risk factors of lung cancer would reduce premature mortality by 28.2% for men and by 23.6% for women compared to the BAU scenario in 2030. These findings suggest that it is more cost-effective to control risk factors for cancers with tendencies toward worse conditions.

Through modeling, our estimates also illustrated significant discrepancies in premature mortality reduction across various risk factor control targets. In parallel with the previous study conducted by Li et al [17], improving physical inactivity resulted in minimal reduction in premature cancer mortality, which might be largely because physical inactivity was only causally related to two cancer sites (ie, colorectal cancer and breast cancer). Another explanation may be that the mild reduction of 10% in physical inactivity was not sufficient to produce a significant reduction in premature cancer mortality in 2030. A similar result was also seen for high red meat intake, which was only causally associated with breast and colorectal cancers. In comparison, smoking is causally associated with a wide range of cancers and showed the largest effect in reducing premature cancer mortality by smoking control. As the largest tobacco producer and consumer globally, China has enacted a set of antismoking policies and regulations after signing the WHO’s Framework Convention on Tobacco Control in 2003 [27], which include public smoking bans, a rise in tobacco taxes, and warnings on the cigarette pack. The serial national surveys on smoking showed that the smoking prevalence in China had been declining steadily in recent years, although remaining at a high plateau among men [28]. Hunan Province has a consistently higher smoking prevalence than the national average level [29], possibly due to a later implementation of smoking restrictions. Recently, Hunan Province launched a series of feasible and effective smoking restriction policies, which have greatly reduced the smoking rate. Several rounds of CCDRFS surveys have also indicated a favorably moderate decrease of the smoking prevalence in Hunan, which may partly account for the relatively lower benefit in mortality reduction through the control of smoking than the control of high BMI and diabetes that have been rising steadily in Hunan. In addition, due to the especially low smoking prevalence among women in Hunan, the WHO target of a 30% reduction may produce much less benefit for women than that among men.

The past decade has also seen significant decreases in the mean population salt intake and prevalence of harmful alcohol use in Hunan, both of which led to more favorable trends than those of the WHO targets. Of note, the prevalence of current alcohol use was still maintained at a high level and was much higher in men than women, especially for hazardous and harmful alcohol use. The high alcohol use prevalence among men in China may be explained by the traditional Chinese culture that encourages drinking as a socially acceptable way to show their dominant positions in society. Disturbingly, there was a substantial increase in alcohol use following the rapid economic transition in China [30]; yet, the current legislation on alcohol use only focuses on problematic alcohol use such as drunk driving, rather than on alcohol use per se. Evidence has shown that alcohol use is the fourth-leading modifiable risk factor that contributes to cancer mortality among Chinese men [6], and abstinence from alcohol would increase life expectancy by 0.77 years [31]. These findings suggest that more effective measures are warranted to reduce alcohol use. The WHO has recommended some cost-effective alcohol prevention measures named “best buys,” which included increasing taxes, enforcing bans on alcohol advertising, and restricting the availability of alcohol [32]. With respect to high salt intake, studies have shown that the daily salt intake in China had been decreasing slowly, which was largely attributed to the government’s multicomponent strategies, including labeling, media campaigns, and voluntary reformulation of the salt industry, among others [33,34]. However, the mean salt intake in 2019 was approximately 9.3 grams per day [35], which was still much higher than the WHO’s recommended level of 6.0 grams per day. Hence, there is still room for improvement in reducing the salt intake among Chinese residents.

Apart from the above risk factors, whether SDG target 3.4 can be realized in 2030 largely depends on the high-impact factors, including diabetes, high BMI, and insufficient intake of fruits and vegetables. China has the largest population of individuals with diabetes in the world, and the prevalence of diabetes has been sharply increasing in recent decades in China, including in Hunan Province [36]. Without proper measures to control blood glucose, China will continue to have the largest population of diabetes in 2030 with a predicted number of 140 million [37]. Previous studies discovered that high concentrations of glucose could provide steady energy for the growth of tumor cells [38,39], and thus diabetes was identified as an independent risk factor for several cancer types [40]. It was estimated that achieving the risk control target for diabetes in 2030 would avoid 57,400 deaths from NCDs in China [17], and our study also predicted notable benefits in premature cancer mortality reduction from the control of diabetes. In China, a range of programs such as primary diabetic health care have been promoted to curb the rapid rise in diabetes [41]. However, due to the imbalanced regional economic development, the capacity of primary medical services is relatively backward in the central and western regions of China, resulting in a lack of standardized diabetic management and low screening rates of diabetes-related complications [42]. Thus, there is still a long way to go to meet the commitments of the UN agenda.

Overweight and obesity prevalence among adolescents and adults has been increasing steadily in China, including Hunan Province, for the past two decades [35]. This is largely attributable to high-calorie dietary habits and reduced physical activity. Researchers have projected that the prevalence of overweight and obesity, if not controlled effectively, might reach 65.3% in adults and 31.8% in adolescents by 2030, with more than 800 million people reaching the overweight and obese categories in China [43]. The government has made great efforts to prevent overweight and obesity, including several school-based programs (such as the Healthy Children Action Plan and National Nutrition Campus Program) and community-based programs (such as the National Healthy Lifestyle Action) to promote healthy diet and exercise among the population [43]. However, existing policies on overweight and obesity are still fragmented in China; thus, coordinated and multisectoral strategies are needed to reach the target of halting the rise in overweight and obesity by 2030.

Fruit and vegetable intake is an indispensable part of a healthy diet. Studies have shown that the average daily intake of fruit in Hunan Province has been increasing steadily in recent years, while the intake of vegetables has been declining [44,45], which reflects a remarkable transition in dietary structure. Despite these trends, the proportions of adults who reached the recommended levels of Chinese dietary guidelines for both fruit and vegetable intake remained at low levels. Globally, inadequate consumption of fruits and vegetables has been validated to contribute to a large portion of the cancer burden in many countries such as the United Kingdom, Japan, and China [3,6,46]. In China, an unhealthy diet including low fruit and vegetable intake has accounted for more than 10% of DALYs [3]. Dietary guidelines with different recommendations have been developed worldwide to tackle the problem. The WHO recommends a total consumption of at least 400 grams per day of fruits and vegetables for adults, while in China the recommended level is 500 grams per day. Although the Global NCD Action Plan has included the prevalence of inadequate intake of fruits and vegetables as a monitoring indicator, no specific target has been set by the WHO to date. In our study, a 30% reduction for inadequate fruit and vegetable intake displayed relatively high benefits in cancer mortality reduction, further supporting the beneficial role of dietary risk control in achieving SDG target 3.4. Currently, health education with a focus on children is the major approach for promoting a healthy diet in China, which indeed has increased people’s awareness [47]. Nevertheless, given that other factors such as availability and prices may also influence eating behavior, there is still much work to be done.

We also validated our projection with other methods or assumptions. In the baseline scenario, where all risk factors continue their current trends, the number of premature deaths from cancers increased from 65,443 in 2017 to 97,787 in 2030. Given that approximately 4 million deaths are expected to occur in 2030 in China [48], an increase of only 32,344 premature deaths in such a populous province may be underestimated. Nevertheless, we further estimated the total number of cancer deaths for all ages and found that the value increased from 111,719 in 2017 to 235,578 in 2030 (see Multimedia Appendix 5), which indicated that people older than 70 years represented the major group of cancer deaths. In addition, assuming that cancer mortality trends continued to 2030 with constant change, we validated the predicted deaths in the BAU scenario using the proportional change model, which showed similar results with only a 3.6% difference in total cancer deaths and a 1.6% difference in overall premature mortality (see Multimedia Appendix 6). Therefore, we believe our projections to be credible.

### Limitations

Some limitations of our work sƒhould be considered. First, Hunan launched its cancer registry program in 2009, and the work at the early stages might be imperfect with low population coverage. Even by the end of 2020, the cancer registration in Hunan had only covered approximately 70% of the population, without achieving full coverage, which may to some extent cause a certain bias in the estimation of actual cancer deaths. Nevertheless, with the efforts of local governments, the coverage of cancer registration has rapidly expanded and the data quality has steadily improved in recent years. Some of the data were even cited in monographs of the International Agency for Research on Cancer. In addition, our data for risk factors were drawn from the CCDRFS survey; therefore, all limitations in estimates of levels in the CCDRFS study apply to this analysis.

Second, we used RR estimates primarily from the GBD series and the CUP Expert Report due to the lack of high-quality meta-analyses and prospective cohort studies in China, which may make our results statistically unstable. However, with more and more large cohort studies being carried out in China, more reliable RRs for China could be available in future studies. Third, due to a lack of dynamic monitoring data, some region-specific risk factors such as betel quid, which is classified as a class 1 carcinogen and has a high prevalence in Hunan, has not been included in this analysis. Considering that the Chinese government has made great efforts on sales restriction and increasing the public awareness of its harm in recent years, the prevalence of betel quid chewing in Hunan may decline steadily; thus, modeling without consideration of betel quid may lead to overestimation of future cancer mortality. Furthermore, there are potential interactions among the selected risk factors; however, due to the absence of information on most interactions, we simply calculated their combined effects on cancers based on the assumption of independence, which may lead to some uncertainties in our results. Hence, solid evidence–based joint RRs on cancers are warranted for future studies.

Fourth, we did not investigate the impact of population aging on premature cancer mortality due to insufficient technological conditions and time. In further studies, we will try to examine the fractions and trends attributable to population aging and its interactions with various risk factors on premature cancer mortality. Fifth, since the current health outcome reflects the cumulative effect of past exposures, risk factors such as smoking and alcohol were subdivided by duration and amount whenever possible. However, no lag effect was considered when data on specific information were unavailable. Nevertheless, calculations of PAFs in our study referred to the comparative risk assessment model from the latest GBD series, and the results were similar to those of previously published literature [6].

### Conclusions

In summary, this modeling study illustrates that the absolute burden of premature deaths due to cancers will continue to increase over the next dozen years in the Hunan province of China. Notable health gains could be achieved by addressing unhealthy risk factors for cancers. However, existing targets on related risk factors are not sufficient, particularly in men, to achieve the one-third reduction goal in premature cancer mortality. More aggressive risk targets based on local conditions are urgently needed.

## Appendix

Multimedia Appendix 1 Details on risk factors and cancer sites.

Multimedia Appendix 2 Risk factor exposure estimation.

Multimedia Appendix 3 Analysis methods.

Multimedia Appendix 4 Scenario projections for each cancer by gender.

Multimedia Appendix 5 Death projection for people of all ages.

Multimedia Appendix 6 Comparison between the baseline scenario and proportional change model.

## Abbreviations

AAPC: average annual percentage change
BAU: business as usual
CCDRFS: Chinese Chronic Disease and Risk Factor Surveillance
CUP: Continuous Update Project
DALY: disability-adjusted life year
GBD: global burden of disease
NCD: noncommunicable disease
NPCR: National Program of Cancer Registries
PAF: population-attributable fraction
PM2.5: fine particulate matter
RR: relative risk
SDG: Sustainable Development Goal
UN: United Nations
WHO: World Health Organization

## Data Availability

The data will be available from the corresponding author on request.

## References

[1] Cancer. World Health Organization.

[2] China: Globocan 2020. Global Cancer Observatory.

[3] GBD 2019 Risk Factors Collaborators (2020). Global burden of 87 risk factors in 204 countries and territories, 1990-2019: a systematic analysis for the Global Burden of Disease Study 2019. Lancet.

[4] Pearce A, Sharp L, Hanly P, Barchuk A, Bray F, de Camargo Cancela M, Gupta P, Meheus F, Qiao Y, Sitas F, Wang S, Soerjomataram I (2018). Productivity losses due to premature mortality from cancer in Brazil, Russia, India, China, and South Africa (BRICS): A population-based comparison. Cancer Epidemiol.

[5] Hofmarcher T, Lindgren P, Wilking N, Jönsson B (2020). The cost of cancer in Europe 2018. Eur J Cancer.

[6] Chen W, Xia C, Zheng R, Zhou M, Lin C, Zeng H, Zhang S, Wang L, Yang Z, Sun K, Li H, Brown MD, Islami F, Bray F, Jemal A, He J (2019). Disparities by province, age, and sex in site-specific cancer burden attributable to 23 potentially modifiable risk factors in China: a comparative risk assessment. Lancet Glob Health.

[7] Transforming our world: the 2030 agenda for sustainable development. United Nations.

[8] Tan X, Liu X, Shao H (2017). Healthy China 2030: a vision for health care. Value Health Reg Issues.

[9] The plan for "Healthy China 2030". CPC Central Committee, State Council.

[10] Zou Y, Liao X, Xu K, Xiao H, Hu Y, Shi Z (2022). Cancer incidence and mortality in Hunan Cancer Registration Areas in 2018. China Cancer.

[11] Zhou M, Wang H, Zeng X, Yin P, Zhu J, Chen W, Li X, Wang L, Wang L, Liu Y, Liu J, Zhang M, Qi J, Yu S, Afshin A, Gakidou E, Glenn S, Krish VS, Miller-Petrie MK, Mountjoy-Venning WC, Mullany EC, Redford SB, Liu H, Naghavi M, Hay SI, Wang L, Murray CJL, Liang X (2019). Mortality, morbidity, and risk factors in China and its provinces, 1990-2017: a systematic analysis for the Global Burden of Disease Study 2017. Lancet.

[12] Hu Y, Zhong R, Li H, Zou Y (2020). Effects of betel quid, smoking and alcohol on oral cancer risk: a case-control study in Hunan Province, China. Subst Use Misuse.

[13] Huang W, Zhu S, Zou Y, Shi Z, Xu K (2017). Incidence and mortality of oral cancer in registered regions of Hunan in 2009–2015. China Cancer.

[14] Global action plan for the prevention and control of noncommunicable diseases 2013-2020. World Health Organization.

[15] Kontis V, Mathers CD, Rehm J, Stevens GA, Shield KD, Bonita R, Riley LM, Poznyak V, Beaglehole R, Ezzati M (2014). Contribution of six risk factors to achieving the 25×25 non-communicable disease mortality reduction target: a modelling study. Lancet.

[16] Su S, Lee W, Chen T, Wang H, Su T, Jeng J, Tu Y, Liao S, Lu T, Chien K (2017). An evaluation of the 25 by 25 goal for premature cardiovascular disease mortality in Taiwan: an age-period-cohort analysis, population attributable fraction and national population-based study. Heart Asia.

[17] Li Y, Zeng X, Liu J, Liu Y, Liu S, Yin P, Qi J, Zhao Z, Yu S, Hu Y, He G, Lopez AD, Gao GF, Wang L, Zhou M (2017). Can China achieve a one-third reduction in premature mortality from non-communicable diseases by 2030?. BMC Med.

[18] Roth GA, Nguyen G, Forouzanfar MH, Mokdad AH, Naghavi M, Murray CJ (2015). Estimates of global and regional premature cardiovascular mortality in 2025. Circulation.

[19] Diet, activity and cancer. World Cancer Research Fund.

[20] Xiao Y (2018). Hunan cancer registry annual report.

[21] Chen W, Zheng R, Baade PD, Zhang S, Zeng H, Bray F, Jemal A, Yu XQ, He J (2016). Cancer statistics in China, 2015. CA Cancer J Clin.

[22] Li Y, Wang L, Jiang Y, Zhang M, Wang L (2013). Risk factors for noncommunicable chronic diseases in women in China: surveillance efforts. Bull World Health Organ.

[23] WHO global air quality guidelines: particulate matter (‎PM2.5 and PM10)‎, ozone, nitrogen dioxide, sulfur dioxide and carbon monoxide. World Health Organization.

[24] Global status report on noncommunicable diseases 2014. World Health Organization.

[25] Ezzati M, Lopez AD, Rodgers AA, Murray CJL Comparative quantification of health risks : global and regional burden of disease attributable to selected major risk factors. World Health Organization.

[26] Chen Y, Guo F, Wang J, Cai W, Wang C, Wang K (2020). Provincial and gridded population projection for China under shared socioeconomic pathways from 2010 to 2100. Sci Data.

[27] Hu T, Lee AH, Mao Z (2013). WHO Framework Convention on Tobacco Control in China: barriers, challenges and recommendations. Glob Health Promot.

[28] China reported health hazards of smoking 2020. National Health Commission of the PRC.

[29] Wang M, Luo X, Xu S, Liu W, Ding F, Zhang X, Wang L, Liu J, Hu J, Wang W (2019). Trends in smoking prevalence and implication for chronic diseases in China: serial national cross-sectional surveys from 2003 to 2013. Lancet Respir Med.

[30] Manthey J, Shield K, Rylett M, Hasan O, Probst C, Rehm J (2019). Global alcohol exposure between 1990 and 2017 and forecasts until 2030: a modelling study. Lancet.

[31] Jiang Y, Liu S, Ji N, Zeng X, Liu Y, Zhang M, Wang LM, Li YC, Zhou MG (2018). Deaths attributable to alcohol use and its impact on life expectancy in China, 2013. Zhonghua Liu Xing Bing Xue Za Zhi.

[32] Xu Q, Zhou M, Jin D, Zeng X, Qi J, Yin L, Liu Y, Yin L, Huang Y (2020). Projection of premature mortality from noncommunicable diseases for 2025: a model based study from Hunan Province, China, 1990-2016. PeerJ.

[33] Tan M, He FJ, Wang C, MacGregor GA (2019). Twenty-four-hour urinary sodium and potassium excretion in China: a systematic review and meta-analysis. J Am Heart Assoc.

[34] Hyseni L, Elliot-Green A, Lloyd-Williams F, Kypridemos C, O'Flaherty M, McGill R, Orton L, Bromley H, Cappuccio FP, Capewell S (2017). Systematic review of dietary salt reduction policies: evidence for an effectiveness hierarchy?. PLoS One.

[35] National Health Commission of the People's Republic of China (2020). Report on Chinese residents' chronic diseases and nutrition 2020. Acta Nutrimenta Sinica.

[36] Hu C, Jia W (2018). Diabetes in China: epidemiology and genetic risk factors and their clinical utility in personalized medication. Diabetes.

[37] Saeedi P, Petersohn I, Salpea P, Malanda B, Karuranga S, Unwin N, Colagiuri S, Guariguata L, Motala AA, Ogurtsova K, Shaw JE, Bright D, Williams R, IDF Diabetes Atlas Committee (2019). Global and regional diabetes prevalence estimates for 2019 and projections for 2030 and 2045: Results from the International Diabetes Federation Diabetes Atlas, 9 edition. Diabetes Res Clin Pract.

[38] Blanc-Lapierre A, Spence A, Karakiewicz PI, Aprikian A, Saad F, Parent M (2015). Metabolic syndrome and prostate cancer risk in a population-based case-control study in Montreal, Canada. BMC Public Health.

[39] Klement RJ, Kämmerer U (2011). Is there a role for carbohydrate restriction in the treatment and prevention of cancer?. Nutr Metab.

[40] Huang Y, Cai X, Qiu M, Chen P, Tang H, Hu Y, Huang Y (2014). Prediabetes and the risk of cancer: a meta-analysis. Diabetologia.

[41] Shi R, Guo X, Zhang Q (2021). Current status and outlook of diabetes self-management education and support in Chinese adults with type 2 diabetes. Chin J Diabetes Mellitus.

[42] Cai C, Jia W (2018). Community healthcare for diabetes in China. Sci Sin Vitae.

[43] Wang Y, Zhao L, Gao L, Pan A, Xue H (2021). Health policy and public health implications of obesity in China. Lancet Diabetes Endocrinol.

[44] Fu Z, Liu J, Liu H, Jin D (2014). Dietary patterns of urban residents from 1982 to 2012 in Hunan. Zhong Nan Da Xue Xue Bao Yi Xue Ban.

[45] Xiao Y, Su C, Ouyang Y, Zhang B (2015). Trends of vegetables and fruits consumption among Chinese adults aged 18 to 44 years old from 1991 to 2011. Zhonghua Liu Xing Bing Xue Za Zhi.

[46] Whiteman DC, Wilson LF (2016). The fractions of cancer attributable to modifiable factors: a global review. Cancer Epidemiol.

[47] Di Sebastiano KM, Murthy G, Campbell KL, Desroches S, Murphy RA (2019). Nutrition and cancer prevention: why is the evidence lost in translation?. Adv Nutr.

[48] Cao W, Chen H, Yu Y, Li N, Cheng WQ (2021). Changing profiles of cancer burden worldwide and in China: a secondary analysis of the global cancer statistics 2020. Chinese Med J.

