# Supplementary material for: Projection of Premature Cancer Mortality in Hunan, China, Through 2030: Modeling Study
Source: JMIR Public Health Surveill. 2023 Mar 6;9:e43967. doi: 10.2196/43967 (PMC10028508; doi:10.2196/43967)
Supplement: Multimedia Appendix 1 [file publichealth_v9i1e43967_app1.docx]

**Multimedia Appendix 1: Details on risk factors and cancer sites**

| **Risk factor** | **Exposure Metric** | **Definition of theoretical minimum risk exposure** | **Disease outcome (ICD-10)** |
| --- | --- | --- | --- |
| **Smoking** | Never-smoker;  Ex-smoker;  Current smoker: light (<15 cigarettes per day); moderate (15-24 cigarettes per day); heavy (≥25 cigarettes per day) | Never smoking | Oral cavity cancer (C00-C08); nasopharynx cancer (C11); esophageal cancer (C15); liver cancer (C22); stomach cancer (C16); lung cancer (C33-C34). |
| **Alcohol use** | Abstainer;  Category I: for females 0-19.99 g pure alcohol daily; for males 0-39.99 g pure alcohol daily.  Category II: for females 20-39.99 g pure alcohol daily; for males 40-59.99 g pure alcohol daily.  Category III: for females 40 g pure alcohol and above; for males 60 g pure alcohol and above. | No alcohol intake | Oral cavity cancer (C00-C08); nasopharynx cancer (C11);  esophageal cancer (C15); liver cancer (C22); stomach cancer (C16); breast cancer (C50); Colorectum cancer (C18-C21) |
| **Physical inactivity** | Level 0: < 600 MET-min/week (inactive);  Level 1: 600-3999 MET-min/week (low-active);  Level 2: 4000-7999 MET-min/week (moderately-active);  Level 3: ≤ 8;000 MET-min/week (highly active) | 600 MET-min/week | Colorectal cancer (C18-C21); breast cancer (C50); |
| **High BMI** | Body mass index (kg/m2) | 22 kg/m2 (SD 1) | Oral cavity cancer (C00-C08); esophageal cancer (C15); colorectum cancer (C18-C21); liver cancer (C22); pancreatic cancer (C25); prostate cancer (C61); breast cancer (C50) |
| **Low vegetable intake** | Vegetable intake per day | 400g (SD 30) intake per day for adults | Lip and oral cavity cancer (C00-C08); nasopharynx cancer (C11); esophagus cancer (C15); tracheal; bronchus; and lung cancer (C33-C34); breast cancer (C50) |
| **Low fruit intake** | Fruit intake per day | 300g (SD 30) intake per day for adults | Esophageal cancer (C15); tracheal; bronchus; and lung cancer (C33-C34); lip and oral cavity cancer (C00-C08); nasopharynx cancer (C11) |
| **High red meat intake** | Red meat intake per day | 18-27g intake per day for adults | Colorectum cancer (C18-C21); breast cancer (C50) |
| **High salt intake** | Salt intake per day | 1-5g intake per day for adults | Stomach cancer (C16) |
| **Diabetes** | Yes/No | 4.8-5.4 mmol/L of fasting glucose | Colorectum cancer (C18-C21); Pancreatic cancer (C25); breast cancer(C50); liver cancer (C22); tracheal; bronchus; and lung cancer (C33-C34) |
| **PM2.5** | Estimated annual average particulate matter concentration for particles with aerodynamic diameters <2.5 microns | 2.4-5.9µg/m3 | Tracheal; bronchus; and lung cancer (C33-C34) |
