# Supplementary material for: Projection of Premature Cancer Mortality in Hunan, China, Through 2030: Modeling Study
Source: JMIR Public Health Surveill. 2023 Mar 6;9:e43967. doi: 10.2196/43967 (PMC10028508; doi:10.2196/43967)
Supplement: Multimedia Appendix 2 [file publichealth_v9i1e43967_app2.docx]

**Multimedia Appendix 2: Risk factor exposure estimation**

| **Risk factor** | **Exposure Metric** | **Men** | | | **Women** | | |
| --- | --- | --- | --- | --- | --- | --- | --- |
|  |  | **2009** | **2017** | **2030*** | **2009** | **2017** | **2030*** |
| Smoking（%） | | | | | | | |
|  | Never-smoker | 25.78 | 29.86 | 35.43 | 96.81 | 97.87 | 98.68 |
|  | Ex-smoker | 8.84 | 8.68 | 8.04 | 0.82 | 0.39 | 0.13 |
|  | Current light smoker | 18.25 | 21.37 | 25.77 | 1.29 | 1.13 | 0.92 |
|  | Current moderate smoker | 33.19 | 30.45 | 25.47 | 0.96 | 0.54 | 0.23 |
|  | Current heavy smoker | 13.94 | 9.65 | 5.29 | 0.12 | 0.08 | 0.04 |
| Alcohol drinking（%） | | | | | | | |
|  | Abstainer | 46.87 | 50.6 | 56.1 | 86.43 | 87.76 | 86.89 |
|  | Category I | 44.64 | 41.85 | 37.55 | 13.21 | 11.25 | 8.56 |
|  | Category II | 3.93 | 3.82 | 3.62 | 0.17 | 0.48 | 2.2 |
|  | Category III | 4.56 | 3.73 | 2.73 | 0.18 | 0.51 | 2.35 |
| Physical inactivity（%） | | | | | | | |
|  | Level 0 | 17.05 | 23.03 | 33.91 | 8.07 | 14.79 | 30.52 |
|  | Level 1 | 38.19 | 32.15 | 23.29 | 50.35 | 43.55 | 29.14 |
|  | Level 2 | 18.59 | 16.57 | 13.08 | 28.32 | 22.77 | 13.65 |
|  | Level 3 | 26.17 | 28.25 | 29.72 | 13.27 | 18.89 | 26.69 |
| Fasting glucose（%） | | | | | | | |
|  | Diabetic | 7.00 | 10.85 | 20.94 | 7.22 | 9.28 | 13.52 |
| BMI (kg/m^2^, mean (SD)) | | | | | | | |
|  |  | 23.11(3.35) | 23.95(3.96) | 25.27(3.69) | 22.78(3.46) | 23.32(3.45) | 24.15(3.69) |
| Vegetable intake（g/d, median (P25, P75)） | | | | | | | |
|  |  | 296.85(287.96, 306.50) | 339.43(322.93, 359.17) | 415.02(383.50, 455.62) | 287.16(277.86, 295.61) | 337.49(322.66, 354.46) | 430.00(403.78, 465.44) |
| Fruit intake（g/d, median (P25, P75)） | | | | | | | |
|  |  | 56.22(52.07, 60.49) | 63.59(55.02, 75.33) | 76.50(59.77, 104.68) | 74.29(70.50, 78.77) | 103.88(95.00, 113.84) | 171.78(148.58, 197.78) |
| Red meat（g/d, median (P25, P75)） | | | | | | | |
|  |  | 118.38(112.91, 124.44) | 152.33(139.78, 167.71) | 222.35(192.54, 262.40) | 86.03(81.86, 90.29) | 103.04(96.19, 109.95) | 135.06(122.54, 147.76) |
| Salt intake（g/d, median (P25, P75)） | | | | | | | |
|  |  | 11.77(11.16, 12.56) | 7.64(7.31, 7.99) | 4.00(3.88, 4.05) | 10.82(10.46, 11.27) | 7.50(7.17, 7.86) | 4.33(4.07, 4.58) |
| PM2.5(µg/m3, mean (SD)) | | | | | | | |
|  |  | 53.4（10.56） | 35.61（9.54） | 17.24（13.16） | 53.4（10.56） | 35.61（9.54） | 17.24（13.16） |

* Projection based on the assumption that past trends between 2009 and 2017 would continue to 2030.
