# Supplementary material for: Projection of Premature Cancer Mortality in Hunan, China, Through 2030: Modeling Study
Source: JMIR Public Health Surveill. 2023 Mar 6;9:e43967. doi: 10.2196/43967 (PMC10028508; doi:10.2196/43967)
Supplement: Multimedia Appendix 3 [file publichealth_v9i1e43967_app3.docx]

**Multimedia Appendix 3: Analysis methods**

This section provides additional information on the methods used in this analysis.

To estimate the number of cancer deaths in Hunan between 2009 and 2017, we first extracted the representative age-, sex-, and cause-specific death data and corresponding rates for all cancers and subcategories from local population-based cancer registries, along with annual sex-specific populations between 2009 and 2017 from Hunan Statistical Yearbook-2020, an officially statistical publication reflecting comprehensively the economic and social development of Hunan. Then the age structure of Hunan from the 2010 national population census was used to estimate age-, sex-, and year-specific numbers, which were ultimately applied for evaluation of cancer deaths throughout the whole province.

The Chinese Chronic Disease and Risk Factor Surveillance (CCDRFS) adopts a multistage stratified cluster sampling method to select participants among permanent residents over 18 years old. And respondents receive a set of standard questionnaire interviews, physical examinations, and biological sampling, in which anthropometric and biochemical data as well as self-reported information on smoking status, alcohol consumption, dietary habits, etc. are collected. In this study, we extracted information on smoking, alcohol consumption, physical activity, BMI, blood glucose, vegetable and fruit intake, red meat intake, and salt intake for calculation. The annual average PM2.5 concentrations were retrieved from the Atmospheric Composition Analysis Group (ACAG), Saint Louis University, which is available at: <https://sites.wustl.edu/acag/datasets/surface-pm2-5/>.

For categorical risk factors like smoking, alcohol consumption, physical activity, etc., the exposure prevalence of each category was calculated for different genders and age groups, and for continuous variables like BMI, fruits, and vegetables, the exposure levels were calculated.

1. Attribution analysis:

According to the comparative risk assessment (CRA) theory of global burden of disease (GBD) study, we used a counterfactual analysis to assess the contribution of certain risk factors to a summary burden of cancer sites. The attributable burden (AD) of certain cancers due to the risk factor was given as AD=PAF×TD, where TD was the total burden of certain cancers.

For continuous risk factors, the PAF was given in the following generalized form:

PAF=$\frac{\int_{x=0}^{m} \mathrm{RR}\left( x \right)P1\left( x \right)dx-\int_{x=0}^{m} \mathrm{RR}\left( x \right)P2\left( x \right)\mathrm{dx}}{\int_{x=0}^{m} \mathrm{RR}\left( x \right)P1\left( x \right)\mathrm{dx}}$

where RR(x) is the RR for exposure level x, P1(x) is the population distribution of exposure, P2(x) is the counterfactual distribution of exposure, and m is the maximum exposure level. We set P2(x) to the theoretical minimum risk exposure adopted by the GBD study, which was reported in Table S1.

For categorical exposure variables, we used the following formula to estimate PAFs:

PAF=$\frac{\sum_{i=1}^{n} P_{i}(\mathrm{RR}_{i}-1)}{\sum_{i=1}^{n} P_{i}\left( \mathrm{RR}_{i}-1 \right)+1}$

where RRi is the RR at exposure level i, P1(x) is the exposure prevalence, and n is the maximum exposure category.

For the burden attributable to clusters of risk factors, we computed the combined PAFs according to the following formula under the assumption of independent exposures and effect:

PAF=$1-\prod_{j=1}^{J} (1-\mathrm{PAF}_{j})$

where j referred to jth risk factor.

2. Prediction analysis:

First, we used the PAF to divide annual deaths into two parts that were attributable or unattributable to the specified risk factors. Second, we projected the unattributable deaths in 2030 using the proportional change model, assuming that changes in this part would continue the trends observed between 2009 and 2017 with a constant annual change rate. And risk factors in baseline scenarios were projected in the same manner.

$\mathrm{Exposure}_{year+1}=\mathrm{exposure}_{\mathrm{year}}\times exp(\frac{\mathrm{In}\left( \frac{\mathrm{exposure}_{2017}}{\mathrm{exposure}_{2009}} \right)}{2017-2009})$

With projected exposures in 2030 and corresponding RRs, we estimated the joint PAFs in 2030 for each risk-outcome pair in different scenarios. The total deaths in 2030 for cancers were then projected by the following formula:

${Total deaths}_{2030}$=${unattributable deaths}_{2030}/(1-\mathrm{PAF}_{2030})$

Premature mortality for cancers under each scenario was projected for 2030. Premature cancer mortality was defined as the probability of dying from cancers between the ages of 30 and 70 years. and was estimated using age-specific death rates (in 5-year age groups between 30 and 70 years) with the following formulas.

${}_{5}{Mx=\frac{Total deaths from cancers between exact age x and exact age x+5}{Total population between exact age x and exact age x+5}}$

For each five-year age group, the probability of death from cancers (_5_q_x_) was calculated：

${}_{5}{q_{x}=\frac{{}_{5}{M_{x}}\times5}{1+{}_{5}{M_{x}\times2.5}}}$

The premature mortality between 30 and 70 years was calculated last:

${}_{40}{q_{30}=1-\prod_{x=30}^{65}\left( 1-{}_{5}{q_{x}} \right)}$

The sex- and age-specific population in 2030 was projected by Yidan Chen, etc [26].
