# Supplementary material for: Projection of Premature Cancer Mortality in Hunan, China, Through 2030: Modeling Study
Source: JMIR Public Health Surveill. 2023 Mar 6;9:e43967. doi: 10.2196/43967 (PMC10028508; doi:10.2196/43967)
Supplement: Multimedia Appendix 4 [file publichealth_v9i1e43967_app4.docx]

# Multimedia Appendix 4: Scenario projections for each cancer by sex

A1. Lung cancer for the whole population

A2. Lung cancer for men

A3. Lung cancer for women

B1. Gastric cancer for the whole population

B2. Gastric cancer for men

B3. Gastric cancer for women

C1. Liver cancer for the whole population

C2. Liver cancer for men

C3. Liver cancer for women

D1. Colon and rectum cancer for the whole population

D2. Colon and rectum cancer for men

D3. Colon and rectum cancer for women

E1. Esophageal cancer for the whole population

E2. Esophageal cancer for men

E3. Esophageal cancer for women

F1. Pancreatic cancer for the whole population

F2. Pancreatic cancer for men

F3. Pancreatic cancer for women

G1. Nasopharynx cancer for the whole population

G2. Nasopharynx cancer for men

G3. Nasopharynx cancer for women

H1. Oral cavity cancer for the whole population

H2. Oral cavity cancer for men

H3. Oral cavity cancer for women

I. Prostate cancer for men

J. Breast cancer for women
