# Supplementary material for: Projection of Premature Cancer Mortality in Hunan, China, Through 2030: Modeling Study
Source: JMIR Public Health Surveill. 2023 Mar 6;9:e43967. doi: 10.2196/43967 (PMC10028508; doi:10.2196/43967)
Supplement: Multimedia Appendix 6 [file publichealth_v9i1e43967_app6.docx]

**Multimedia Appendix 6: Comparison between the baseline scenario and proportional change model**

| **Gender** | **Disease** | **Premature deaths** | | **Premature mortality (%)** | |
| --- | --- | --- | --- | --- | --- |
|  |  | **if risk factor trends continue** | **Proportional Change model** | **if risk factor trends continue** | **Proportional Change model** |
| **Men** | Total | 71920 | 70477 | 14.24 | 12.72 |
|  | Lung cancer | 22599 | 22857 | 4.54 | 4.59 |
|  | Gastric cancer | 2290 | 2055 | 0.46 | 0.42 |
|  | Liver cancer | 12360 | 10686 | 2.61 | 2.28 |
|  | Colon and rectum cancer | 7539 | 7124 | 1.60 | 1.51 |
|  | Esophageal cancer | 5916 | 5784 | 1.35 | 1.32 |
|  | Pancreatic cancer | 1414 | 1386 | 0.28 | 0.27 |
|  | Nasopharynx cancer | 2852 | 3184 | 0.63 | 0.70 |
|  | Oral cavity cancer | 7255 | 7646 | 1.71 | 1.80 |
|  | Prostate cancer | 2504 | 2563 | 0.52 | 0.53 |
|  | Other cancers | 7193 | 7193 | 1.46 | 1.46 |
| **Women** | Total | 25867 | 23828 | 5.11 | 3.50 |
|  | Lung cancer | 6738 | 6294 | 1.38 | 1.29 |
|  | Gastric cancer | 763 | 507 | 0.17 | 0.12 |
|  | Liver cancer | 3185 | 2764 | 0.59 | 0.51 |
|  | Colon and rectum cancer | 4803 | 4212 | 0.92 | 0.81 |
|  | Esophageal cancer | 61 | 44 | 0.01 | 0.01 |
|  | Pancreatic cancer | 271 | 275 | 0.06 | 0.06 |
|  | Nasopharynx cancer | 400 | 399 | 0.09 | 0.09 |
|  | Oral cavity cancer | 215 | 201 | 0.04 | 0.04 |
|  | Breast cancer | 3185 | 2886 | 0.69 | 0.63 |
|  | Other cancers | 6245 | 6245 | 1.27 | 1.27 |
| **Both** | Total | 97787 | 94305 | 9.74 | 8.17 |
|  | Lung cancer | 29337 | 29151 | 2.96 | 2.94 |
|  | Gastric cancer | 3053 | 2562 | 0.32 | 0.26 |
|  | Liver cancer | 15545 | 13450 | 1.59 | 1.38 |
|  | Colon and rectum cancer | 12342 | 11335 | 1.25 | 1.15 |
|  | Esophageal cancer | 5977 | 5828 | 0.68 | 0.66 |
|  | Pancreatic cancer | 1685 | 1661 | 0.17 | 0.16 |
|  | Nasopharynx cancer | 3252 | 3584 | 0.35 | 0.39 |
|  | Oral cavity cancer | 7470 | 7847 | 0.87 | 0.92 |
|  | Prostate cancer | 2504 | 2563 | 0.52 | 0.53 |
|  | Breast cancer | 3185 | 2886 | 0.69 | 0.63 |
|  | Other cancers | 13438 | 13438 | 1.36 | 1.36 |
