# Supplementary material for: Projection of Premature Cancer Mortality in Hunan, China, Through 2030: Modeling Study
Source: JMIR Public Health Surveill. 2023 Mar 6;9:e43967. doi: 10.2196/43967 (PMC10028508; doi:10.2196/43967)
Supplement: Multimedia Appendix 5 [file publichealth_v9i1e43967_app5.docx]

**Multimedia Appendix 5: Death projection for people of all ages**

| **Gender** | **Disease** | **2013** | **2030** | **Percent change (%)** |
| --- | --- | --- | --- | --- |
| **Men** | Total | 63640 | 153937 | 141.89 |
|  | Lung cancer | 23029 | 49237 | 113.81 |
|  | Gastric cancer | 4901 | 5261 | 7.34 |
|  | Liver cancer | 11159 | 17874 | 60.18 |
|  | Colon and rectum cancer | 4712 | 14222 | 201.80 |
|  | Esophageal cancer | 2556 | 7530 | 194.57 |
|  | Pancreatic cancer | 1123 | 4762 | 324.22 |
|  | Nasopharynx cancer | 1889 | 3687 | 95.14 |
|  | Oral cavity cancer | 745 | 7900 | 960.90 |
|  | Prostate cancer | 1011 | 12331 | 1119.21 |
|  | Other cancers | 12503 | 31135 | 149.01 |
| **Women** | Total | 32533 | 81641 | 150.95 |
|  | Lung cancer | 7130 | 24134 | 238.51 |
|  | Gastric cancer | 2633 | 8981 | 241.04 |
|  | Liver cancer | 3522 | 12421 | 252.67 |
|  | Colon and rectum cancer | 2858 | 9823 | 243.67 |
|  | Esophageal cancer | 578 | 1173 | 102.92 |
|  | Pancreatic cancer | 760 | 3036 | 299.44 |
|  | Nasopharynx cancer | 685 | 660 | -3.67 |
|  | Oral cavity cancer | 214 | 540 | 152.22 |
|  | Breast cancer | 2505 | 4481 | 78.88 |
|  | Other cancers | 11647 | 16394 | 40.76 |
| **Both** | Total | 96173 | 235578 | 144.95 |
|  | Lung cancer | 30158 | 73371 | 143.29 |
|  | Gastric cancer | 7535 | 14242 | 89.02 |
|  | Liver cancer | 14681 | 30295 | 106.36 |
|  | Colon and rectum cancer | 7571 | 24044 | 217.59 |
|  | Esophageal cancer | 3134 | 8703 | 177.67 |
|  | Pancreatic cancer | 1883 | 7798 | 314.22 |
|  | Nasopharynx cancer | 2575 | 4347 | 68.85 |
|  | Oral cavity cancer | 959 | 8439 | 780.21 |
|  | Prostate cancer | 1011 | 12331 | 1119.21 |
|  | Breast cancer | 2505 | 4481 | 78.88 |
|  | Other cancers | 24151 | 47529 | 96.80 |
